# Low-Power Distributed Data Flow Anomaly-Monitoring Technology for Industrial Internet of Things

**DOI:** 10.3390/s19122804

**Published:** 2019-06-22

**Authors:** Weihong Han, Zhihong Tian, Wei Shi, Zizhong Huang, Shudong Li

**Affiliations:** 1 Cyberspace Institute of Advanced Technology, Guangzhou University, Guangzhou 510006, China; hanweihong@gzhu.edu.cn (W.H.); lishudong@gzhu.edu.cn (S.L.); 2 School of Information Technology, Carleton University, Ottawa, ON K7L 3R5, Canada; weishi@cunet.carleton.ca; 3 Computer School, National University of Defense Technology, Changsha 410073, China; 13800419839@139.com

**Keywords:** anomaly monitoring, data flow, industrial internet of things, low power consumption, wireless sensor network

## Abstract

In recent years, the industrial use of the internet of things (IoT) has been constantly growing and is now widespread. Wireless sensor networks (WSNs) are a fundamental technology that has enabled such prevalent adoption of IoT in industry. WSNs can connect IoT sensors and monitor the working conditions of such sensors and of the overall environment, as well as detect unexpected system events in a timely and accurate manner. Monitoring large amounts of unstructured data generated by IoT devices and collected by the big-data analytics systems is a challenging task. Furthermore, detecting anomalies within the vast amount of data collected in real time by a centralized monitoring system is an even bigger challenge. In the context of the industrial use of the IoT, solutions for monitoring anomalies in distributed data flow need to be explored. In this paper, a low-power distributed data flow anomaly-monitoring model (LP-DDAM) is proposed to mitigate the communication overhead problem. As the data flow monitoring system is only interested in anomalies, which are rare, and the relationship among objects in terms of the size of their attribute values remains stable within any specific period of time, LP-DDAM integrates multiple objects as a complete set for processing, makes full use of the relationship among the objects, selects only one “representative” object for continuous monitoring, establishes certain constraints to ensure correctness, and reduces communication overheads by maintaining the overheads of constraints in exchange for a reduction in the number of monitored objects. Experiments on real data sets show that LP-DDAM can reduce communication overheads by approximately 70% when compared to an equivalent method that continuously monitors all objects under the same conditions.

## 1. Introduction

The wireless sensor network in the front-end of the industrial internet of things (IoT) often consists of a large number of distributed sensors, where the monitored data are continuously transmitted to the master control node in the form of data flow. The monitoring of the overall state of the system is often determined collectively by the state of each sensor [1,2]. This process is described as monitoring of anomalies in distributed data flows. For example, for applications such as asset and inventory management in the industrial IoT [3,4], or monitoring of power consumption in industrial production, each sensor node monitors data in real time and continuously aggregates the monitored data to a master monitoring node, which will then identify whether the overall situation of the system has exceeded a previously defined security threshold. Due to the largely spread and often hazardous sensor locations in various applications and the cost limitation of sensor nodes in wireless sensor networks, power consumption is often the main factor limiting the performance of an IoT system. Therefore, it is of great significance to study the low-power anomaly-monitoring solutions in distributed data flows for the industrial IoTs [5,6].

To provide a solution on monitoring anomalies in distributed data flows in the industrial IoT when the focus is on reducing the communication overheads and power consumption, a low-power distributed data flow anomaly-monitoring model (LP-DDAM) implementing an algorithm that integrates multiple sets of monitored objects into a single and complete set after fully considering the relationships among objects is presented. In the industrial IoT, sensors are often divided into regions, such as sensors in the warehouse management and monitoring system. Whole system includes multiple warehouses distributed throughout the country, and there are several sensors distributed in each warehouse. The traditional method is that each sensor communicates with the system’s master control node in real time to monitor anomalies. The LP-DDAM method can treat multiple monitored objects in the same region as a whole, and makes full use of the relationship among objects, so that only one representative object needs to communicate with the master control node. The communication overhead is greatly reduced, and the power consumption of sensors except the representative object is also reduced.

LP-DDAM method selects the objects with the largest global values as a set of representative objects among all the objects that may or may not exceed the predefined threshold value. Local adjustment on each representative object factor is applied and local constraints are set up to ensure the correctness of the continuous monitoring process. The continuous monitoring of multiple objects is replaced by monitoring the representative object as well as the local constraints. Only when local constraints are broken, communication and parameter adjustments are needed to reconstruct the local monitoring process. The proposed algorithms, adjustment process and adjustment factor allocation strategy are described in detail in Section 3. In Section 4, the correctness of the algorithm is proved and the extended application of this algorithm in a variety of situations is studied. Experiments on real data sets are explained in Section 5. The results show that the use of the LP-DDAM model can effectively reduce communication overheads in monitoring of distributed data flows to 70% under the same conditions. We conclude the paper and point out future directions in Section 6.

## 2. Background

Data flow anomaly monitoring has been a very active research field since it was proposed [7,8,9,10], and has been widely used in network security, industrial control, online monitoring and real-time online services [11,12,13,14,15].

For distributed data flow anomaly monitoring, Dilman et al. [16] first put forward the idea of reducing communication overheads. By combining event reporting with rotational monitoring, they proposed two methods, i.e., the simple-value method and the simple-rate method, to reduce communication overheads. In their methods, the remote node no longer monitored the local value but the changes of the local value to reduce the communication overheads. Kale et al. [17] used the trigger method to study threshold monitoring, proposed five evaluation parameters to measure the performance of the algorithm and provided many potential solutions, such as the statistical probability-based method, the global distributed hash table method, and others. Sun et al. [18], to meet the needs of distributed data flow monitoring in smart cities in the future, studied the topic of distributed data flow monitoring from two different perspectives: improving communication efficiency and data privacy protection. In their research, each remote node was assigned multiple local thresholds, representing different “grades”. The centralized nodes only needed to know the “grade” where each local value was located to estimate the global value that would satisfy the accuracy requirement, according to its upper and lower bounds, thus considerably reducing the communication overheads. Macker et al. [19] studied the top-K problem in distributed data flow monitoring and used the filter to reduce the communication between distributed data flow monitoring nodes and primary nodes. Wang et al. [20] studied the anomaly detection method of distributed data flow in vehicle-mounted communication systems, and adopted the method of pre-learning to predict the status of subsequent data flows to reduce communication overheads and improve accuracy. Sadeghioon et al. [21] studied real-time anomaly detection based on temperature and pressure data from real-time sensors in water pipeline monitoring systems. They proposed the methods of dividing and adjusting local thresholds in distributed data flow monitoring, including uniform division, proportional division, static thresholds and dynamic thresholds. They verified the effectiveness of their proposed methods through a large number of experimental analyses.

## 3. Low-Power Distributed Dataflow Anomaly Monitoring Model

### 3.1. Model Description

In the industrial IoT, the problem of distributed data flow anomaly monitoring is described as follows: the system consists of *m* monitored objects *O_i_* (*i* = 1, …, *m*), *n* remote monitoring nodes *N_j_* (*j* = 1, …, *n*) and a centralized node *N_0_*. *V_i,j_* represents the monitored local value of the object *O_i_* on the remote monitoring node *N_j_*, so the global value of each object is $V_{i}=\sum_{j}V_{i,j}$. Each remote node monitors the local data flow *S_j_*, and the new tuple <*O_i_*, *N_j_*, *t*, *V_i,j,t_*> causes the continuous change of *V_i,j_* as *V_i,j_* = *V_i,j_* + *V_i,j,t_*. The system monitors the anomaly continuously on *N_0_* by identifying which object’s global value exceeds the threshold *T* in real time. For each object *O_i_* whose value V_i_ exceeds the threshold *T_i_*, the approximate value *V_i_’*, which satisfies the pre-specified precision constraint, is obtained.

When monitoring anomalies in distributed data flows, users are often interested in anomalies (i.e., those objects that exceed the threshold) only, while data indicating normal behaviors do not need to be recorded constantly. Therefore, the definition of monitoring of anomalies in distributed data flows in a broader sense is given as follows:

**Definition** **1.** *Approximate threshold monitoring. For any given threshold T and precision constraint parameter δ, the approximate monitoring value V_i_’ for object O_i_ satisfies the following formulas:*

(1) $$\begin{array}{l} 0\leq V_{i}^{\prime }<T\;\mathrm{when}\;V_{i}<T\\ \left|V_{i}-V_{i}^{\prime }\right|\leq \delta \;\mathrm{when}\;V_{i}\geq T \end{array}$$

*where*$V_{i}=\sum_{j}V_{j}$.

That is, when a global value *V_i_ for object O_i_* is small, the user is not interested in its specific value other than that it is below the threshold. Only when the global value exceeds the threshold *T*, its specific value within precision *δ* is required to be tracked.

Assessment of the values of the threshold relies on global information. However, each monitoring node can only observe its local data. In industrial IoT scenarios, the remote sensor nodes for distributed monitoring are limited by their environment, so their power consumption and communication overheads must be considered. Obviously, if all information about node changes is transmitted to *N_0_*, the monitoring will create vast amount of network overheads. Monitoring by the periodic snapshot acquisition method usually presents periodic outbursts of oscillation of network traffic as well, and a compromise between snapshot frequency and communication overheads must be considered: excessive high frequency will generate large network overheads, while a frequency that is too low will lead to a delay or even loss of reporting on abnormal events. Therefore, the challenge is to reduce communication overheads while still ensuring the correctness of the results and timely response to abnormal events.

### 3.2. Overview of the Model 

First, the LP-DDAM model we propose is based on the following two observations:

Observation 1. The relationships between the global values *V_i_* for different objects are stable during a short period of time.

Observation 2. In most cases, the global value *V_i_* of an object does not exceed a threshold *T*.

Observation 1 indicates that in the industrial IoT, the relationship between the global values of each monitored object will not change dramatically during any given short period of time. Here is an example on monitoring of power consumption in industrial production [22]: for a given period of time, the power consumption of the lathe producing part 1 on a certain node is always higher than that of the lathe producing part 2. Observation 2 suggests that there exists an anomaly when the monitored event value exceeds a given threshold. Therefore, in general, an object stays longer in the normal state than in the abnormal state, in other words, at any given point in time, majority of the monitored objects are in normal state while minority are in abnormal state (when object value exceeds a given threshold). Based on these two observations, a method to reduce power consumption and communication overheads is proposed when monitoring multiple distributed data flows simultaneously. The overall idea is as follows:

(1) For all objects that exceed each of their threshold *T_i_*, the existing uniform threshold assignment (UTA) is used to continuously track approximate values satisfying accuracy constraints, to ensure that the monitored value of each object satisfies the requirements set forth in Definition 1.
(2) For most objects that do not exceed their corresponding *T_i_*, only the object *O_max_* with the largest global value is selected as the representative value for continuous tracking.
(3) Through the “adjustment factor” parameter, we adjust the local value of the object *O_max_* to be the largest on each remote node to ensure that other objects can be represented by *O_max_*, that is, if *O_max_* does not exceed the threshold, other objects do not exceed the threshold either.
(4) Continuous monitoring of multiple objects is transformed into monitoring of *O_max_* keeping its local maximum constraints. Communication is required only when the constraints are no longer satisfied due to the arrival of new data, thus reducing the communication overheads.

**Definition** **2.** *Representative object. $\forall O_{j}\neq O_{i}$, $V_{i}\geq V_{j}$, that is, if O_i_ has the largest global value, it is called the representative object, which is represented by O_max_.*

**Definition** **3.** *Adjustment factors. An adjustment factor ε_i,j_ is assigned to each node N_j_ (j = 0, …, n) for each object O_i_, satisfying the following constraints:*

(2) $$\begin{array}{l} (1)\;\forall O_{i},\sum_{j=0}^{n}\varepsilon_{i,j}=0\\ (2)\;\forall N_{j}(1\leq j\leq n),and\;\forall O_{i}\neq O_{\max},\;V_{\max,j}+\varepsilon_{\max,j}\geq V_{i,j}+\varepsilon_{i,j} \end{array}$$

Constraint (1) requires the sum of all adjustment factors of each object to be zero, so it will not affect the correctness of the global value. Constraint (2) requires the representative object to have the largest local value on each remote node after the adjustment factors are added.

The symbols used here and their meanings are shown in Table 1.

### 3.3. Model Description

#### 3.3.1. Framework of Algorithms for the Model

The low-power distributed data flow anomaly-monitoring model (LP-DDAM) is shown as Algorithm 1. For all object sets *OT* that exceed the threshold *T*, the existing UTA method is used for continuous approximate monitoring. Therefore, for the sake of simplicity of description, it is assumed that the set of objects that do not exceed the threshold is *U*, and only the unified monitoring method of these objects is considered. This concept is presented below as the LP-DDAM algorithm. The core of the LP-DDAM algorithm is to make the local value *V_max,j_* + *ε_max,j_* of the representative object *O_max_* appear to be the maximum on each monitoring node by adjusting the factor. Therefore, as long as this constraint is satisfied, the method of continuous monitoring of *O_max_* can be adopted to replace that of monitoring all objects: as long as the global value of *O_max_* does not exceed *T,* other objects will certainly not exceed *T*. When *O_max_* exceeds *T* or when the global value of an object that previously exceeded the threshold is less than *T* at a certain point of time, the LP-DDAM algorithm needs to be invoked again to select the representative object and allocate adjustment factors.

**Algorithm 1.** Low-power distributed data flow anomaly-monitoring model (LP-DDAM). 1*N_0_* obtains the initial value *V_i,j_* of each object, and selects the object *O_max_* with the largest global value as the representative for continuous monitoring. Then, the reallocation algorithm (defined below in Section 3.3.3) is used to assign the adjustment factors *ε_i,j_* on each node *N_j_* (*j* = 0, …, *n*) for each object *O_i_* (*i* =1, …, *m*) in *U*, and then they are sent to the corresponding *N_j_*.2Each monitoring node *N_j_* (*j* = 1, …, *n*) monitors the local data flow *S_j_* separately:3 While (1)4   Read the data in *S_j_* <*O_i_*, *N_j_*, *t*, *V_i,j,t_*>5   *V_i,j_* = *V_i,j_* + *V_i,j,t_*6   If $\exists O_{c}\in U,O_{c}\neq O_{\max}$, *V_max,j_* + *ε_max,j_* ≤ *V_c,j_* + *ε_c,j_*7    Use the “Adjustment algorithm” to adjust the system8If the centralized node N_0_ finds that V_max_ exceeds the threshold, then we set *OT* = *OT* + {*O_max_*}, and *U* = *U* − {*O_max_*}, and then the LP-DDAM algorithm is again used.9If the object that previously exceeded the threshold is below the threshold at a certain time, the third step in the resolution process is called for adjustment.

#### 3.3.2. Adjustment Process

At a certain point, when the remote node *N_c_* finds that local constraints are broken, it needs to call the “Adjustment process” for adjust the system (Lines 6 and 7 of the LP-DDAM algorithm). Definitions of several terms are given next.

**Definition** **4.** *Conflict sets. Conflict sets are sets of objects that violate constraints, that is, $C=\{O_{i}|V_{i,c}+\varepsilon_{i,c}>V_{\max,c}+\varepsilon_{\max,c}\}$ including all objects with local values greater than O_max_ on the N_c_ node.*

**Definition** **5.** *Lower bound. The conflict set is on the lower bound $B_{j}=\max\{V_{i,j}+\varepsilon_{i,j}|O_{i}\in U-C-O_{\max}\}$ on the node N_j_.*

The adjustment process is described as Algorithm 2. When allocating adjustment factors, to avoid a global adjustment every time the constraint is broken, we do not allocate all “surpluses” to remote nodes; instead, a part is reserved on *N_0_*, marked as *ε_i,0_*, and the lower boundary $B_{0}=\max\{\varepsilon_{i,0}|O_{i}\in U-C-O_{\max}\}$ on *N_0_* is defined. When *N_c_* sends the reconstruction constraint request, the first step is to determine whether the adjustment can be completed through *ε_i,0_*. If it can, the adjustment only occurs between *N_0_* and *N_c_*; otherwise, it is necessary to obtain the values of the related objects from other remote monitoring nodes for global allocation and adjustment. 

The lower bound *B_j_* is a parameter introduced to reduce the traffic of each adjustment. It represents the upper bound of local values of objects other than conflict sets C and *O_max_* on the node *N_j_*. By using this parameter, the transfer of a large number of local values of objects is avoided.

**Algorithm 2.** Adjustment. 1*N_c_* sends reconstruction constraint requests to *N_0_*, including the conflict set *C*, the monitoring values of objects in *C* on *N_c_*, *V_max,c_* and the lower bound *B_c_*.2*N_0_* carries out a validity test: If $\forall O_{c}\in C$,$V_{\max,c}+\varepsilon_{\max,0}+\varepsilon_{\max,c}\geq V_{c,c}+\varepsilon_{c,0}+\varepsilon_{c,c}$, then the validity test is successful. Call the “reallocation algorithm” to recalculate the adjustment factors of *N_c_* and *N_0_*, and then the new adjustment factors are sent to *N_c_*. At this point, the resolution process ends. If the validity test fails, the third step is executed.3*N_0_* obtains the monitored values of objects and *O_max_* in set *C* from each node $N_{j}(1\leq j\leq n,\;j\neq c)$ and the lower bound *B_j_*, and then identifies the new representative object *O’_max_* according to the aggregated values of objects. Finally, it calls the reallocation function to recalculate the adjustment factors of all nodes, and sends *O’_max_* and new adjustment factors to each monitoring node.

#### 3.3.3. Adjustment Factor Allocation

The LP-DDAM algorithm calls the reallocation function to calculate the adjustment factor in the initialization stage, as well as the second and third stages in the resolution process, but the adjustment factor is calculated only between *N_c_* and *N_0_* in the second stage of the resolution process, while the adjustment factors on all nodes are calculated in the initialization stage and the third stage of the resolution process. The concepts of partially aggregated values of participated nodes set *N* and objects on *N* are defined next.

**Definition** **6.** *Participated nodes set. In the second stage of the resolution process, the participated nodes set is N = {N_c_,N_0_}; in other cases, N = {N_i_|i = 0, …, n}.*

**Definition** **7.** *Partial aggregation values. The partial aggregation value V_iN_ of object O_i_ on N, and the partial aggregation value B_N_ on the lower bound are defined as follows:*

(3) $$\begin{array}{l} V_{iN}=\varepsilon_{i,0}+\sum_{1\leq j\leq n,N_{j}\in N}\left(V_{i,j}+\varepsilon_{i,j}\right)\\ B_{N}=\sum_{0\leq j\leq n,N_{j}\in N}B_{j} \end{array}$$

**Definition** **8.** *Allocation factors. When calculating the adjustment factors, an allocation factor F_j_ is assigned to each node, representing the allocation strategy of the adjustment factor. The allocation factor satisfies the following constraints:*

(1) $0\leq F_{j}\leq 1$;
(2) If $N_{j}\notin N$, *F_j_* = 0;
(3) $\sum_{j=0}^{n}F_{j}=1$

The description of the process of adjustment factor allocation is shown in Algorithm 3. It can be seen that the reallocation process first approximatively calculates the “surplus” of each object according to the lower bound, and then distributes the “surplus” between the remote node and the centralized node according to a certain strategy, so that the difference between the adjusted object value and the lower bound is *F_j_λ_i_*. The different assignment of allocation factors reflects the difference of adjustment factors in allocation strategies. Generally speaking, the allocation factor *F_0_* on *N_0_* needs to be specified separately to allocate large “surplus” for local adjustment in phase 2 of the resolution process. The remaining “surplus” can be allocated among *N_j_* (*j* = 1, …, *n*) nodes according to a certain strategy. The choice of *F_0_* needs to consider the balance between the adjustment frequency and the communication required for each adjustment: the larger *F_0_* is, the more adjustments are local adjustments, so the required amount of communication for each adjustment is small. However, the larger the *F_0_*, the stricter the constraints of the remote nodes are, and the easier it is to violate the local constraints, that is, the more frequent the resolution adjustment will be. Conversely, the smaller the *F_0_* is, the smaller is the chance for remote nodes to break constraints, but the greater the possibility of global adjustment in phase 3 of the resolution process, that is, the greater the traffic per adjustment.

**Algorithm 3.** Reallocation **Input:** C, N, {B_j_}, {V_i,j_}, {ε_i,j_}, {F_j_}**Output:** {*ε’_i,j_* }1For each object *O_i_* in $R=C\cup \{O_{\max}\}$, the allocated balance *λ_i_* is calculated as *λ_i_* = *V_iN_* − *B_N_*2For each object *O_i_* in *R*, its new adjustment factor *ε’_i,j_* on each node in *N* is calculated as *ε’_i,j_ = B_j_ − V_i,j_ + F_j_λ_i_*

The allocation of *F_j_* (*j* = 1, …, *n*) should reflect the distribution of data in different nodes. According to the actual situation, we can choose from the following allocation strategies:

1. **Average allocation strategy**. The “surplus” is allocated equally among remote nodes, i.e., *F_j_* = (1 − *F*_0_)/(|*N*| − 1).
2. **Proportional allocation strategy**. The allocation of “surplus” is proportional to the lower bound *B_j_* of node *N_j_*, i.e., *F_j_* = (1 − *F*_0_)*B_j_*/(*B_N_* − *B*_0_).
3. **Inversely proportional allocation strategy**. The allocation of “surplus” is inversely proportional to (*V_max,j_* − *B_j_*), i.e., $F_{j}=(1-F_{0})(\frac{1}{|N|-1}-\frac{V_{\max,j}-B_{j}}{V_{\max,N-\{N_{0}\}}-B_{N}+B_{0}})$.

## 4. Analysis and Extension of the Model

### 4.1. Proof of Correctness of the Proposed Solution

The proof of correctness on the proposed solution is provided below. First, it is assumed that the adjustment calculation takes less time than the tuple arrival interval in the data flow, that is, no new data arrives during the adjustment process. Obviously, the algorithm is correct when all remote nodes satisfy local constraints, so we only need to prove that the two constraints in Definition 3 are still satisfied after the adjustment in the resolution process is completed. This is proved by mathematical induction as follows.

1. LP-DDAM initialization calls only for reallocation adjustment factors. $\forall O_{i}\notin R$, *O_i_* does not participate in the allocation, so *ε_i,j_* = 0, which obviously satisfies the constraint (1). $\forall O_{i}\in R$, according to the reallocation algorithm, we obtain the following equation:

(4) $$\sum_{j=0}^{n}\varepsilon_{i,j}=\sum_{j=0}^{n}B_{j}-V_{i,j}+F_{j}\lambda_{i}=B_{N}-V_{i,N}+\sum_{j=0}^{n}F_{j}\lambda_{i}.$$

According the constraint (3) of Definition 8 and the definition of *λ_i_* in Line 1 of the reallocation algorithm, $\sum_{j=0}^{n}\varepsilon_{i,j}=0$ can be obtained.

$\forall N_{j}(1\leq j\leq n)$, *V_max,j_* + *ε_max,j_* = *B_j_* + *F_j_λ_max_*

$\forall O_{i}\neq O_{\max}$, if $\forall O_{i}\notin R$, then *ε_i,j_* = 0. According to the definition of the lower bound, $V_{i,j}\leq B_{j}$, then $V_{\max,j}+\varepsilon_{\max,j}\geq V_{i,j}+\varepsilon_{i,j}$. $\forall O_{i}\in R$, (*V_max,j_* + *ε_max,j_*) − (*V_i,j_* + *ε_i,j_*) = (*B_j_* + *F_j_λ_max_*) − (*B_j_* + *F_j_λ_i_*) = *F_j_*(*λ_max_* − *λ_i_*) = *F_j_*(*V_max,N_* − *V_i,N_*). From the Definitions 2, 7 and 8, it follows that *F_j_*(*V_max,N_* − *V_i,N_*) ≥ 0, so $V_{\max,j}+\varepsilon_{\max,j}\geq V_{i,j}+\varepsilon_{i,j}$.

Therefore, after LP-DDAM calls the reallocation initialization, the system satisfies the two constraints of Definition 3.

2. If the two constraints of Definition 3 are satisfied on all remote nodes before the local constraints are broken, we then prove that the two constraints are still satisfied after the local constraints are broken and the resolution process is adjusted. 

If the resolution process ends after step 2 is implemented, it means that the representative object *O_max_* has not changed and the adjustment only occurs between *N_0_* and *N_c_*. Therefore, for $\forall N_{j}\neq N_{c}$ and $\forall O_{i}\notin R$ on *N_c_*, the constraints are still satisfied. For $\forall O_{c}\in C$ participating in the adjustment, it satisfies the condition $V_{\max,c}+\varepsilon_{\max,0}+\varepsilon_{\max,c}\geq V_{c,c}+\varepsilon_{c,0}+\varepsilon_{c,c}$. Similarly, to the above proof process, the two constraints of Definition 3 can still be satisfied after adjustment.

If the resolution process executes step 3, according to the definition of the lower bound, *V_max,c_* > *B_c_*. For any other nodes $N_{j}(j\neq c)$ that do not violate local constraints, it is obvious that *V_max,j_* > *B_j_*, so *λ_max_* = *V_max,N_* − *B_N_* > 0. Therefore, whether the new object is the same as the original object or not, the calculated “surplus” based on the lower bound must be positive. The same process can be applied to other proofs. In conclusion, the above method proves the correctness of the algorithms.

### 4.2. Performance Analysis of the Algorithms

The network traffic of the distributed data flow anomaly monitoring depends on the number of communications between remote nodes and centralized nodes and the size of messages in each communication [23]. On one hand, it is related to data distribution and on the other hand, it is closely related to the selection of algorithm’s parameters. Existing algorithms deal with monitoring of multiple objects independently. Although some methods have been adopted to reduce the communication overheads, in general, the communication overheads of these methods are linearly related to the number of objects *m*. However, LP-DDAM only selects a representative object to monitor to adjust the additional cost of constraints in exchange for fewer actual monitored objects, so there is no obvious linear relationship between the traffic and the number of monitored objects. In industrial IoT applications, front-end sensors are often large-scale distributed systems, and the number of sensors is large. Communication overheads of the LP-DDAM algorithm will not increase linearly with the number of sensors, which greatly reduces the system’s traffic. Compared with the existing methods, the LP-DDAM algorithm is also affected by different characteristics of data distribution. LP-DDAM is sensitive to the stability of the relationship between monitored objects, while the existing methods (such as the UTA algorithm) are greatly affected by the changes in the span of the object value itself. Therefore, they can complement each other to a certain extent and meet the monitoring needs of data with different distribution characteristics.

### 4.3. LP-DDAM Model Extension

LP-DDAM assumes that all monitored objects have the same threshold parameter *T*. However, in real-world scenarios, this hypothesis does not necessarily hold in many cases. For such cases, standardization can be carried out, that is, the threshold of all the objects is considered as 1, and the local value *V_i,j_* is converted into *V’_i,j_* = *V_i,j_*/*T_i_*, where *T_i_* is the corresponding threshold of the object *O_i_*. Correspondingly, the data tuple <*O_i_*, *N_j_*, *t*, *V_i,j,t_*> becomes <*O_i_*, *N_j_*, *t*, *V_i,j,t_*/*T_i_*>. Through this standardization change, the LP-DDAM algorithm can deal with the problem of monitoring multiple objects with different thresholds.

If the monitoring value of object *O_max_* on a node drops and leads to the violation of local constraints, the conflict set *C* may be large. In order to reduce the communication overheads during adjustment, the following improvements can be considered. When a remote node *N_j_* receives data <*O_i_*, *N_j_*, *t*, *V_i,j,t_*> and finds that the constraint is broken and |*C*| > *Φ* (*Φ* is threshold set by user), *V_i,j_ = V_i,j_ −* *V_i,j,t_* is ordered to cancel the effect of this data on *V_i,j_*. Then, *N_j_* sends a data <*O_i_*, *N_j_*, *t*, *V_i,j,t_*> to *N_0_*. After *N_0_* receives it, *N_0_* sends data <*O_i_*, *V_i,j,t_*> to other nodes. Each remote node *N_w_* (*w* ≠ *j*) calculates the size of the conflict set |*C_w_*| caused by *V_i,w_* = *V_i,w_* + *V_i,j,t_*, and sends it to *N_0_*. *N_0_* selects the node with the smallest |*C_w_*| value as the node whose constraint is broken to rebuild the constraint.

If the approximation problem in Definition1 is slightly extended as follows,

(5) $$\begin{array}{l} 0\leq V^{\prime }<T\;\mathrm{when}\;V<(1+\delta )T\\ \left|V-V^{\prime }\right|\leq \delta T\;\mathrm{when}\;V\geq (1+\delta )T \end{array}$$

then the local constraints on LP-DDAM remote nodes (i.e., condition 2 in Definition 3) can be further relaxed as follows: *V_i,j_* + *ε_i,j_* ≤ (1 + *δ*)(*V_max,j_* + *ε_max,j_*), that is, the local values of other objects can exceed the local values of *O_max_* within a certain range. This is because monitoring *O_max_* with the UTA method can ensure that *V_max_* < *T*. Therefore $\sum_{j=0}^{n}\left(1+\delta )(V_{\max,j}+\varepsilon_{\max,j}\right)<(1+\delta )T$, that is, the representation of object *O_max_* can still be guaranteed after relaxing the conditions. As long as the representative object does not exceed the threshold and the local constraints are not broken, the global values of other objects will not exceed (1 + *δ*)*T*. Therefore, using this extension of the algorithm can further reduce communication overheads.

## 5. Experimental Analysis

### 5.1. Experimental Data

**Figure 1:**
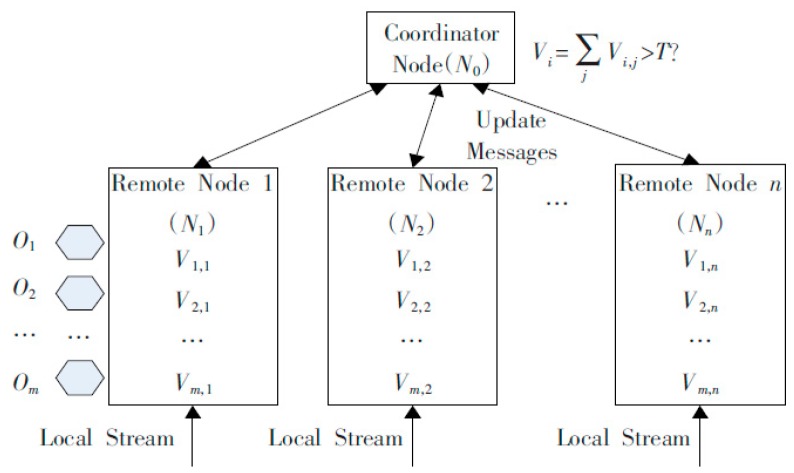
System topology of experiment.

We tested the LP-DDAM algorithm with the industrial IoT monitoring data from Sany Heavy Industry [24]. Sany Heavy Industry is the first enterprise in China that applies the industrial IoT to production process monitoring and equipment management. All large-scale equipment manufactured by Sany Heavy Industry sends its location information, equipment status and other information to the master control platform in real time through a network of sensors. The test data set used in this paper was the data obtained from the equipment power consumption anomaly monitoring system of Sany Heavy Industry. We duplicated the data set of monitored power consumption of 50 kinds of equipment from 10 monitoring nodes in a month, and the system topology is shown in the Figure 1. Among them, *O*_1_~*O_m_* are the monitoring objects. Here we have selected 50 monitoring devices, so *m* = 50. *Node 1~Node n* were the monitoring nodes, and we selected 10 distributed monitoring nodes, so n = 10. The test data was the power consumption and temperature anomaly of the monitoring system, the monitoring interval is 5 s and continuous monitoring was performed for 7 × 24 hour. The equipment monitored was distributed in 10 areas, 50 devices in each area, so the data volume per month was (60 × 60/5) × 24 × 30 × 10 × 50, which was about 260 million. We tested the performance of the LP-DDAM algorithm with this data set.

### 5.2. Experiment Results and Analysis

The purpose of the LP-DDAM technology proposed in this paper was to reduce the communication overheads. Therefore, we tested the influence of various parameters of the LP-DDAM algorithm on the communication overheads and compared the communication overheads with that of the existing algorithms (the classical UTA algorithm was chosen as a comparison).

#### 5.2.1. Influence of Allocation Factor and Allocation Strategy on Communication Overheads

**Figure 2:**
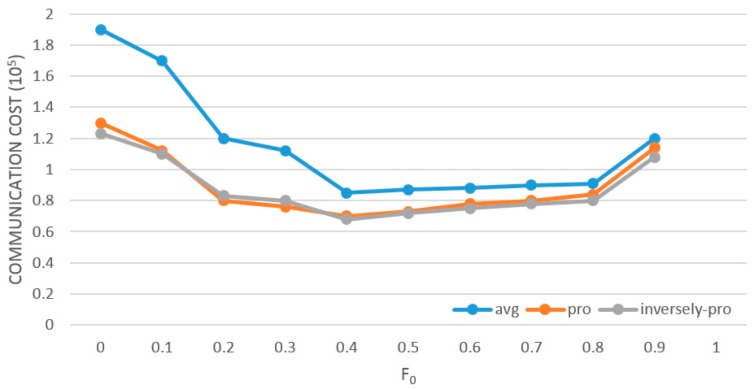
Influence of allocation factor and its allocation strategy on low-power distributed data flow anomaly-monitoring model (LP-DDAM).

The assignment strategy of the allocation factor *F_j_(j =* 0, …, *n)* plays the key role in determining the performance of LP-DDAM in reducing communication overheads. Figure 2 shows the system’s communication overheads (*T* = 2,500,000, *δ* = 0.001) when *F_0_* was pre-allocated with different values and the adjustment factor adopts three strategies: average allocation (avg), proportional allocation (pro) and inversely-proportional allocation (inversely-pro). It shows that the value of *F_0_* had a great impact on the algorithm’s performance in reducing communication overheads. When *F_0_* was between 0.4 and 0.6, there was a good balance between the frequency of violations of local constraints and the cost of each adjustment. In general, the method of allocating adjustment factors proportionally achieves better effect than the average allocation method because they considered the conditions of data distribution on different nodes, and among the methods of allocating adjustment factors proportionally, the inversely-proportional strategy was slightly better than the proportional strategy. For this reason, the follow-up experiments were conducted with the inversely-proportional allocation strategy.

#### 5.2.2. Influence of Allocation Factor and Thresholds on Communication Overheads

**Figure 3:**
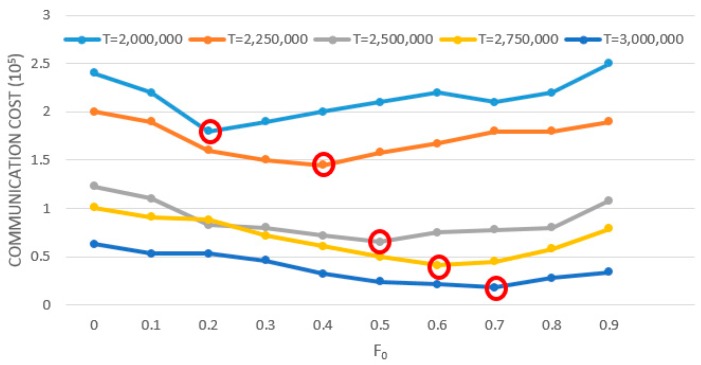
Influence of allocation factor and thresholds on LP-DDAM.

The influence of the value of *F_0_* on the communication overhead of the algorithm was also related to the monitoring threshold of the system. Therefore, we tested the monitoring threshold from T = 2,000,000 to T = 3,000,000. The test results are shown in the Figure 3. It can be seen that the larger the monitoring threshold is, the smaller the communication overhead of the system is, because the chance of breaking constraint becomes smaller. When the value of *F_0_* was large, the effect of reducing the communication overhead will be better. On the contrary, when the monitoring threshold was small and the value of *F_0_* was small, the remote node will have less chance to break the constraint, and its effect will be better. The follow-up experiments were conducted with a fixed T = 2,500,000, and *F_0_* = 0.5.

#### 5.2.3. Change of Communication Overheads with the Number of Monitored Objects

**Figure 4:**
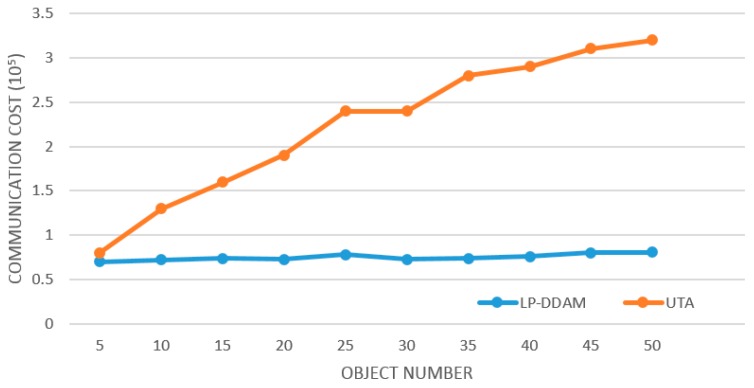
Change of communication overheads with the number of monitored objects.

Figure 4 shows the comparison of traffic between LP-DDAM and UTA (*T* = 2,500,000, *δ* = 0.001) on the monitored data set of Sany Heavy Industry’s equipment power consumption as the number of monitored objects increases. The two algorithms are compared under the same conditions. It is clear that the UTA method needs continuous monitoring of each object, so the traffic increases linearly with the number of monitored objects. However, the LP-DDAM method only monitored the representative object continuously while ensuring that the local constraints are not broken, so the required traffic is not significantly affected by the number of objects. Therefore, in the large-scale multi-object monitoring condition, there was a clear advantage of the LP-DDAM method in reducing communication overheads.

#### 5.2.4. Influence of Error Parameters on Reducing Communication Overheads

**Figure 5:**
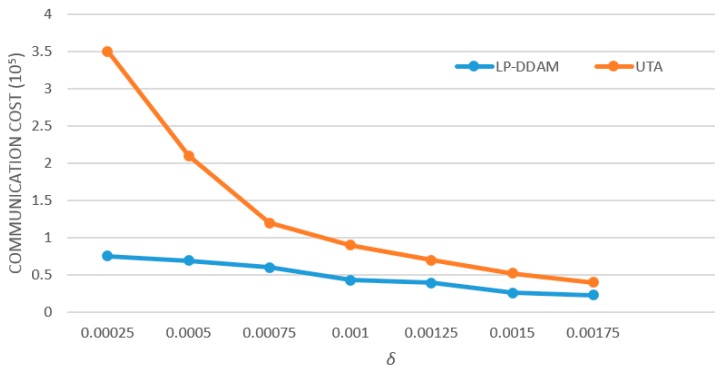
Influence of error parameters on reducing communication overhead.

The influence of accuracy error *δ* on reduction of communication overheads is shown in Figure 5 (*T* = 2,500,000). The two algorithms are compared under the same conditions. The larger the *δ* was, the larger the “grade” width (*δT/M*) used by the UTA method was. Therefore, the smaller the probability of the change of the object’s “grade” was, the smaller the traffic required by the system was. LP-DDAM used the UTA method to process all objects that exceed the threshold of the representative object, so the communication overheads will decrease with the increase of *δ*. However, UTA is more sensitive to the change of *δ*. It can be seen from the figure that when the *δ* value is small, the traffic required by the UTA method increases sharply. In contrast, the LP-DDAM method changes more smoothly, that is, the LP-DDAM method has more obvious advantages in situations with a smaller *δ*.

#### 5.2.5. Changes of Communication Overheads with Threshold Parameters

**Figure 6:**
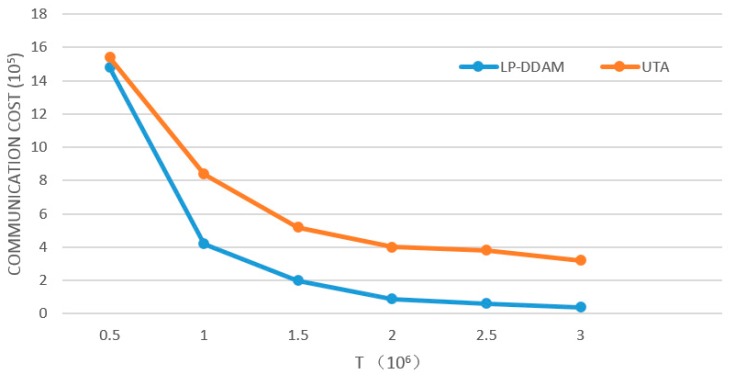
Changes of communication overheads with threshold parameters.

The number of monitored objects is fixed (*M* = 50), and the threshold *T* is changed to change the proportion of objects exceeding the threshold to all objects. The effect of threshold parameters on traffic is analyzed, as shown in Figure 6. The two algorithms are compared under the same conditions. LP-DDAM reduces communication overheads mainly by exploring the relationship between objects below the threshold. With the decrease of *T*, more objects exceed the threshold, and these objects were tracked by the UTA method to obtain approximate solutions to meet the accuracy requirements. In addition, the objects fluctuated repeatedly in the threshold, which called the eighth and ninth lines of the LP-DDAM algorithm to initialize or adjust globally, resulting in larger communication overheads.

Therefore, the lower the value of *T* was, the smaller the advantage the LP-DDAM method had. In extreme cases where all objects exceed the threshold, the LP-DDAM method transformed into the UTA method. Experiments on two datasets showed that when the global values of more than 60% of the objects were below the threshold, the LP-DDAM method needed less than 30% of the traffic of the UTA method.

## 6. Conclusions

To provide a feasible solution on monitoring anomalies in distributed data flows in industrial IoT, we introduce a low-power distributed dataflow anomaly-monitoring model in this paper. The model is conceived under the assumption that (1) the monitoring system is only interested in detecting anomalies, which are rare, and (2) the relationship among the objects regarding the size of their attribute values in real-world practice is usually stable over any given period of time. In the proposed solution, multiple objects are integrated in a single whole set, making full use of the relationship between objects. The model selects only one set of “representative” object for continuous monitoring and establishes certain constraints to ensure correctness, that is, other objects can be represented by this “representative object”, or it is guaranteed that the representative object will always have the largest global value. The communication overheads can be reduced because of the reduction in the number of monitored objects. Experiments on real data sets show that LP-DDAM can reduce communication overheads by about 70% comparing with those achieved by using methods running continuous monitoring of each object under the same conditions.

A consequence of using LP-DDAM to reduce communication overheads is the increased costs on computation and storage. In addition, because of the characteristics of the LP-DDAM method, its performance in reducing communication overheads is conditional and not applicable to all distributed data sets. When there is no obvious relationship among the objects in terms of the size of their global values, the “representative object” will be displaced frequently. When the global values of many objects fluctuate frequently around the threshold due to improper selection of threshold parameters, the LP-DDAM model must undergo a great deal of global adjustments, resulting in extra costs that are likely to outweigh the benefits. Therefore, in some special application scenarios where Observation 1 and Observation 2 described in Section 3.2 do not hold, the LP-DDAM approach to reduce communication overheads is likely to fall short of expectations.

This paper does not discuss the security and trustworthiness of the algorithm. In the future work, we will research on the security and trusty of LP-DDAM algorithm. In addition, when the application scenarios where Observation 1 and Observation 2 described in Section 3.2 do not hold, how to reduce the communication overhead is the further research direction.

## Figures and Tables

**Table 1:** List of symbols.

Symbols	Meaning	Symbols	Meaning
*U*	Universe of data objects	*V_i,j_*	Partial data value of *O_i_* on node *N_j_*
*T*	User-specified threshold	*ε_i,j_*	Adjustment factor for *V_i,j_*
*O_i_*	Data object (*i* = 1, …, *m)*	*N_c_*	Remote node violating the local constrain
*O_max_*	The representative object	*C*	Set of objects violating the local constrain
*N_0_*	Central coordinator node	*R*	Set of nodes participating in resolution
*N_j_*	Remote node *(j =* 1, …, *n)*	*N*	Set of all nodes
*S_j_*	local data flow in *N_j_*	*B_j_*	Border value from node *N_j_*
*V_i_*	Global value for object *O_i_*	*OT*	Set of objects that exceed the threshold T
*δ*	precision constraint parameter	*V′*	approximate monitoring value
